# The lack of homozygotes with a large deletion encompassing *SPAG1* and *POLR2K* in primary ciliary dyskinesia patients suggests the lethal effect of the loss of POLR2K protein

**DOI:** 10.1016/j.gendis.2025.101535

**Published:** 2025-01-20

**Authors:** Alicja Rabiasz, Monika Drobna-Śledzińska, Patrycja Kaźmierczak, Michał Witt, Ewa Ziętkiewicz

**Affiliations:** Institute of Human Genetics, Polish Academy of Sciences, Poznań 60-479, Poland

*SPAG1* (sperm-associated antigen 1) is one of over 50 genes whose pathogenic variants underlie primary ciliary dyskinesia (PCD; OMIM244400), an inherited disorder affecting the function of motile cilia,^1^ highly conserved organelles protruding from the surface of eukaryotic cells. Pathogenic *SPAG1* variants impair the assembly of dynein arms, essential elements of cilia. In a cohort of almost 300 affected individuals with the genetic bases of PCD explained during our long-term studies, pathogenic variants in *SPAG1* were found in 60 unrelated patients, making it one of the most frequently involved genes in the Polish PCD population. Two predominant, previously reported variants^1^^,^^2^ were a nonsense mutation c.2014C>T in exon 16, found in both homozygotes and in compound heterozygotes, and a large 11,973 bp deletion encompassing parts of *SPAG1*, and of the upstream *POLR2K* (RNA polymerase II, I and III subunit K). c.2014C>T was found in 27 homozygotes and 31 compound heterozygotes, of whom 28 had the large deletion as the second allele; the large deletion was found in two more compound heterozygotes, accompanied in trans by rare pathogenic variants (Fig. 1A and Tables S1 and 2). Similar to previous reports,^1^^,^^2^ the large deletion was repetitively found in compound heterozygosity, but no homozygotes were identified.

**Figure 1:**
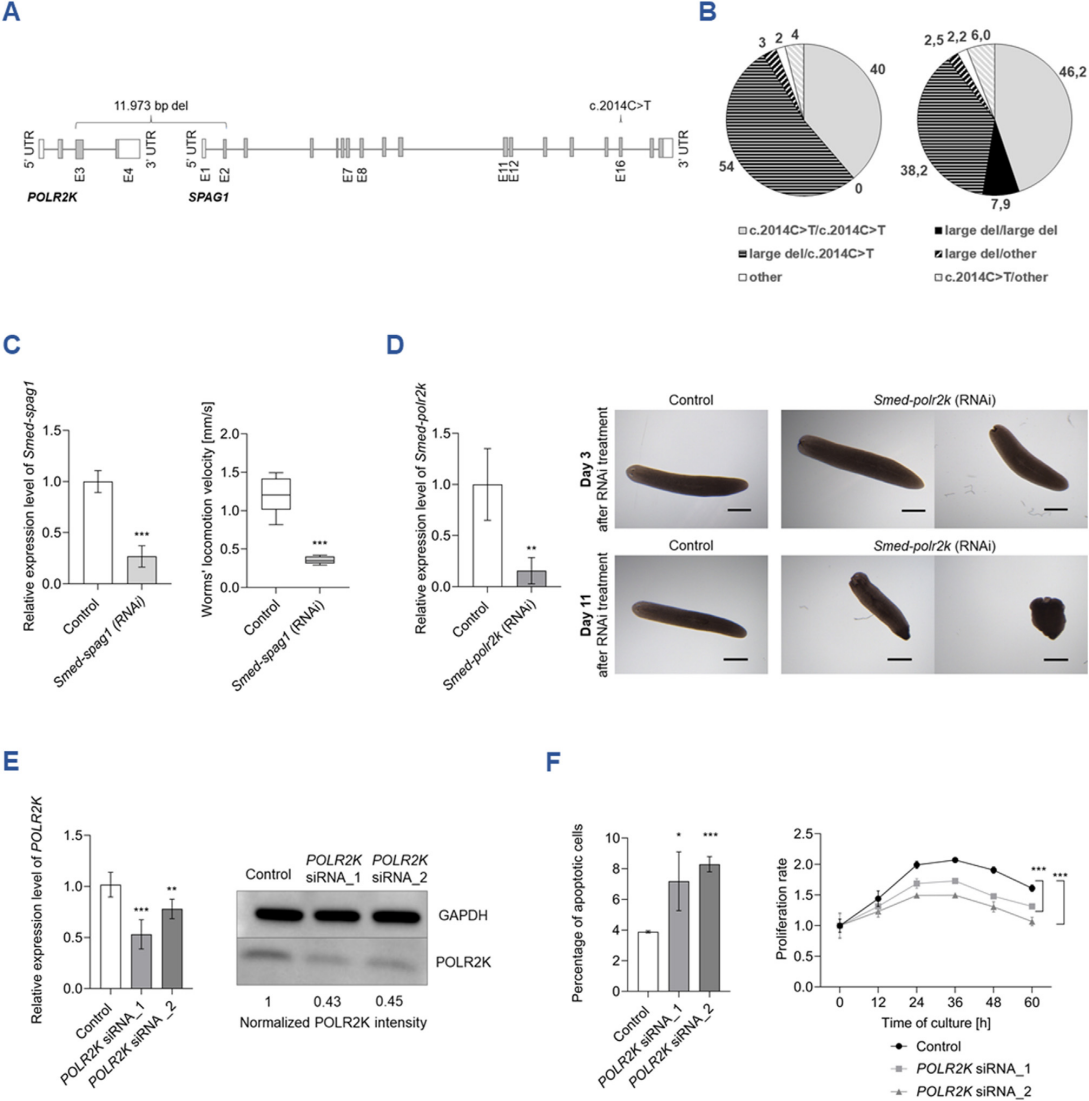
The elucidation of the role of *POLR2K* in the pathogenesis of primary ciliary dyskinesia (PCD). **(A)** Scheme of the localization of pathogenic variants detected in the analyzed group of 60 *SPAG1*-PCD patients. Two frequent deletion are indicated above the gene scheme: 11,973 bp del: a large 11,973 bp deletion [NM_005034.4:c.61 + 201_NM_003114.5:c.140 + 1169]del, encompassing exons 1 and 2 of SPAG1 (sperm-associated antigen 1) and exons 3 and 4 of POLR2K (RNA polymerase II, I and III subunit K) located upstream of SPAG1 in chromosome 8q22.2 (indicated by a bracket); c.2014C>T in exon 16 (NM_003114.5:c.2014C>T; NP_003105.2:p.Gln672∗). *SPAG1* exons with pathogenic variants are indicated by numbers. **(B)** Distribution of *SPAG1* genotypes in the combined group of 103 unrelated Caucasian *SPAG1*-PCD patients. Left panel: The count of observed genotypes. Right panel: The expected number of genotypes calculated assuming Hardy–Weinberg equilibrium. The expected number of the deletion homozygotes in the joint group of 103 *SPAG1*-PCD patients raised to eight; with none observed, the difference between observed and expected was statistically significant (*p* = 0.0066). **(C)** Analysis of the impact of *Smed*-*spag1* knockdown on *S. mediterranea* movement. Left: Effectiveness of the dsRNA-mediated *Smed-spag1* knockdown. DsRNA-mediated knockdown reduced the relative expression level of *Smed-spag1* to 27% compared with control worms treated with dsRNA targeting *eGFP* (measured by quantitative reverse transcription PCR one day after the last administration of dsRNA; *n* = 4; ∗∗∗*p* < 0.001). Right: The speed of planarians' locomotion at day 2 after the last administration of dsRNA (1.189 ± 0.371 and 0.354 ± 0.065 mm/s, in controls and *Smed*-*spag1*-silenced worms, respectively). Plots show the mean value of biological replicates (*n* = 10); error bars indicate standard deviation; ∗∗∗*p* < 0.001. **(D)** Analysis of the impact of *Smed-polr2k* knockdown on *S. mediterranea*'s phenotype. Left: Effectiveness of the dsRNA-mediated *Smed-polr2k* knockdown. Quantitative reverse transcription PCR (measured one day after the last administration of dsRNA) showed a decrease in *Smed-polr2k* expression level to 14% compared with control worms; plots show the mean value of biological replicates (*n* = 4); error bars indicate standard deviation; ∗∗*p* < 0.01. Right: Examples of worm's phenotype abnormalities (deformation of body shape, tissue lysis) after *Smed-polr2k* knockdown, compared with a control (upper and lower panel: day 3 and 11 after last dsRNA feeding, respectively); the defects finally lead to worms' death. The first changes in the worm's phenotype were observed 3 days after the last dsRNA feeding and affected 54% of the worms; it gradually affected all *Smed-polr2k*-silenced worms. Scale bar: 1 mm. (**E**) Efficiency of the siRNAs-mediated *POLR2K* knockdown in human cells, 48 h post transfection. Left: Quantitative reverse transcription PCR analysis of *POLR2K* mRNA level. *POLR2K* siRNA_1 and *POLR2K* siRNA_2 reduced the relative expression level of *POLR2K* to ∼54% and ∼78%, respectively (non-targeting siRNA was used as a negative control). Quantitative reverse transcription PCR plots show the mean value of biological replicates (*n* = 5); error bars indicate standard deviation; ∗∗*p* < 0.01, ∗∗∗*p* < 0.001. Right: Western blot analysis of POLR2K; at the protein level, the knockdown efficiency was comparable for both siRNAs; normalized to GAPDH. **(F)** Analysis of the impact of *POLR2K* knockdown on vital functions in human cells. Left: Apoptosis assay performed using flow cytometry. Percentage of apoptotic cells: 7.2% and 8.3% 48 h after transfection with *POLR2K* siRNA_1 and *POLR2K* siRNA_2, respectively, compared with 3.9% in the negative control. Plots show the mean value of biological replicates (*n* = 3); error bars indicate standard deviation; ∗*p* < 0.05, ∗∗∗*p* < 0.001. Right: Cell proliferation rate (assessed using colorimetric assay) was calculated as the fold change in OD450 for each examined time point in reference to the starting point (0 h); biological replicates, *n* = 3; ∗∗∗*p* < 0.001.

In our group of 60 *SPAG1*-PCD patients, ∼4 homozygotes with the large deletion are expected under Hardy–Weinberg equilibrium; however, none was observed. This difference in the predicted and observed distribution of genotypes was not statistically significant (chi-square, *p* = 0.1663). To achieve the statistical power, we added data for 18 Caucasians reported earlier^1^^,^^2^ (8 compound heterozygotes carrying the large deletion), and for 25 *SPAG1*-PCD German patients (6 c.2014C>T homozygotes and 19 heterozygotes for c.2014C>T and the large deletion; Heymut Omran, personal communication). The expected number of the large deletion homozygotes in the joint group of 103 patients raised to eight (Fig. 1B); with none observed, the unbalanced distribution of the genotypes was statistically significant (*p* = 0.006).

The lack of large deletion homozygotes in the combined group of 103 patients suggested a seriously deleterious effect (presumably lethality) of this genotype. Given that the large deletion encompasses the C-terminal part of *POLR2K* in addition to exons 1 and 2 of *SPAG1*, we hypothesized that this was due to the lack of functional POLR2K rather than SPAG1 protein. *POLR2K* represents one of the smallest subunits of RNA polymerase II, shared with two other RNA polymerases; in consequence, *POLR2K* is involved in the RNA processing machinery and improves RNA polymerase III pre-initiation complex assembly.^3^ To confirm our hypothesis, we examined the effect of RNA interference-mediated knockdown of *POLR2K* at two levels: the whole organism (ciliated flatworm, *Schmidtea mediterranea*), and cellular (human cell line).

*S. mediterranea* is used as a model organism to study the functionality of embryonic-lethal genes in the adult body and cilia-related genes (worms' locomotion depends on motile cilia covering the ventral side of their body). To compare the phenotypes caused by the deficiency of *Smed-spag1* and *Smed-polr2k* in *S. mediterranea*, we first performed a Smed-*spag1* knockdown. The effectiveness of gene silencing was confirmed using quantitative reverse transcription PCR (Fig. 1C-left). The worms upon knockdown displayed no morphological defects, but moved three times slower than controls, using characteristic inchworming (Fig. 1C-right; supplementary movies 1 and 2).

Supplementary video related to this article can be found at https://doi.org/10.1016/j.gendis.2025.101535

The following are the supplementary data related to this article:

VideoS1 2 VideoS1

VideoS2 3 VideoS2

VideoS3 4 VideoS3

In *Smed*-*polr2k-*knockdown worms, the relative expression level was also significantly reduced (Fig. 1D-left). In contrast to *Smed*-*spag1*-silencing, the knockdown of *Smed-polr2k* led to severe defects of planarian bodies, including tissue lysis and head regression, which ultimately led to worms' death (Fig. 1D-right). Furthermore, despite the enormous regenerative capacity of planarians, *Smed-polr2k* deficiency resulted in disruption of the regeneration process (the lack of worms' regrowth after cutting) (Fig. S1). On the other hand, until the point of tissue lysis, no worms' motility impairment was observed (supplementary movie 3), indicating the lack of *Smed-polr2k* involvement in motile cilia function.

To better understand the phenotype observed in worms and to relate the obtained results to humans, the effect of *POLR2K* knockdown on the vital cell functions was assessed in the human HEK293T cell line transfected in parallel with two siRNAs targeting different regions of *POLR2K* exon 3. Quantitative reverse transcription PCR and western blotting confirmed the effectiveness of the gene knockdown at both mRNA and protein levels, respectively (Fig. 1E). *POLR2K* knockdown resulted in a gentle but statistically significant increase in the number of apoptotic cells (Fig. 1F-left). The cell proliferation was significantly decreased in the *POLR2K*-silenced cells compared with the control (Fig. 1F-right).

In the presented study, we examined the hypothesis that the discrepancy between the observed and expected distribution of haplotypes in *SPAG1*-PCD patients (the lack of homozygotes with the large deletion encompassing parts of *SPAG1* and *POLR2K*), reflected a highly deleterious, possibly lethal effect of the loss of a functional POLR2K rather than of SPAG1 protein. A similarly unbalanced distribution of genotypes has been described for *PMM2* gene^4^ (zero observed versus eight expected p.R141H homozygotes in 54 Caucasian patients with carbohydrate-deficient glycoprotein syndrome type 1A). The authors concluded that this distribution was probably caused by selection against the homozygous R141H.^4^ It should be emphasized that the frequency of homozygous pathogenic variants introducing premature STOP codon located in *SPAG1* exon 16 (c.2014C>T) was even higher than expected under equilibrium, indicating the lack of selection against a homozygous deficiency of the wild-type *SPAG1*.

To provide a molecular context to our observation, we analyzed the impact of *POLR2K* silencing on phenotypes at the whole organism (planarians) and cellular levels. The dsRNA-mediated knockdown of *Smed-spag1* in *S. mediterranea* did not affect the viability of the organisms, but as expected, impaired worms' movement, confirming the evolutionary conserved role of *SPAG1* in the motile cilia function (previously demonstrated using a vertebrate model, zebrafish^1^). In contrast, silencing of *Smed*-*polr2k* resulted in a much more severe phenotype and suggested that *Smed-polr2k* played an essential role in maintaining body homeostasis by regulating cell turnover, probably through the impact on planarian stem cell proliferation. Our observations are in line with those in other studies where deleterious effects of other essential gene knockdowns on *S. mediterranea* phenotype have been reported. For example, RNA interference-mediated knockdown of *Argonaute RISC catalytic component 2* (*Smed-ago2*)^5^ led to regeneration defects or tissue regression in worms, ultimately causing their death, demonstrating that *Smed-ago2* is crucial for tissue homeostasis and regeneration and acts through a regulation of adult stem cell proliferation.^5^

Our results of siRNA-mediated *POLR2K* knockdown in the human cell culture shed light on the mechanisms of defects observed in the planarian model. They confirmed the essential role of *POLR2K* in the regulation of vital cell functions, manifested by decreased cell proliferation and increased apoptosis upon its knockdown.

Despite the repetitive reports suggesting *POLR2K's* importance in many cellular processes (additional discussion), no direct experimental proof of phenotype changes after *POLR2K* knockdown has been published. Our findings confirm that *POLR2K* is necessary for the functioning of living organisms and suggest that its influence on cell turnover is exerted through the regulation of cell proliferation and apoptosis. Moreover, our study provides evidence that the essential role of *POLR2K* in vital cell functions is evolutionarily conserved. Finally, these results, together with the earlier studies regarding *POLR2K* as a hub gene involved in many essential interactions, support our starting hypothesis that the lack of homozygotes of the large deletion involving *SPAG1* and *POLR2K* genes is caused by the lethal impact of the POLR2K deficiency; at the same time, it indicates that *POLR2K* is not directly involved in the PCD pathogenesis. Our findings suggest that potential mRNA-based therapy for *SPAG1*-PCD patients with large deletion should be designed to restore SPAG1, and not POLR2K function.

## Ethics declaration

The diagnostics were conducted according to the recommendation of the World Medical Association. Written informed consent was obtained from the participating subjects or their guardians. Procedures representing part of the diagnostic scheme were approved by the institutional committee (the Ethics Committee of the Medical University in Poznań, 960/11, 435/13, and 381/22).

## Funding

This research was funded by the 10.13039/501100004442National Science Centre, Poland (No. 2018/29/N/NZ5/00810 to A.R.; 2018/31/B/NZ2/03248 to E.Z.) and by the Institute of Human Genetics PAS, Poland (Minigrant No. 2021/01 to A.R.).

## CRediT authorship contribution statement

**Alicja Rabiasz:** Writing – review & editing, Writing – original draft, Visualization, Validation, Methodology, Investigation, Funding acquisition, Formal analysis, Data curation, Conceptualization. **Monika Drobna-Śledzińska:** Writing – review & editing, Methodology, Investigation, Formal analysis. **Patrycja Kaźmierczak:** Writing – review & editing, Investigation. **Michał Witt:** Writing – review & editing, Supervision. **Ewa Ziętkiewicz:** Writing – review & editing, Writing – original draft, Supervision, Investigation, Funding acquisition, Formal analysis, Data curation, Conceptualization.

## Data availability

The data that support the findings of this study are available from the corresponding authors upon reasonable request.

## Conflict of interests

The authors declare that they have no known competing financial interests or personal relationships that could have appeared to influence the work reported in this paper.
