# Supplementary material for: The lack of homozygotes with a large deletion encompassing SPAG1 and POLR2K in primary ciliary dyskinesia patients suggests the lethal effect of the loss of POLR2K protein
Source: Genes Dis. 2025 Jan 20;12(6):101535. doi: 10.1016/j.gendis.2025.101535 (PMC12311433; doi:10.1016/j.gendis.2025.101535)
Supplement: Multimedia component 1 [file mmc1.docx]

**Supplementary Materials**

**The lack of homozygotes with large deletion encompassing *SPAG1* and *POLR2K* in primary ciliary dyskinesia patients suggests lethal effect of the loss of POLR2K protein**

**Alicja Rabiasz*, Monika Drobna-Śledzińska, Patrycja Kaźmierczak, Michał Witt^#^, Ewa Ziętkiewicz***

Institute of Human Genetics, Polish Academy of Sciences, 60-479 Poznań, Poland

*Correspondence: Alicja Rabiasz alicja.rabiasz@igcz.poznan.pl, phone: +48 616579253;
Ewa Ziętkiewicz ewa.zietkiewicz@igcz.poznan.pl, phone: +48 616579203

^#^affiliation expired

1. **Additional Background**

Cilia and flagella are highly conserved organelles that form protrusions on the surface of many eukaryotic cells. Mutations in genes encoding ciliary structural proteins or proteins involved in cilia biogenesis affect a variety of tissues and organs of the human body and lead to a wide range of diseases and syndromic disorders named ciliopathies ^1^. Primary ciliary dyskinesia, PCD (OMIM244400) is a key example of a hereditary ciliopathy caused by the dysfunction of motile cilia. Multiple clinical symptoms of PCD (lower and upper respiratory tract distress, hearing impairment, infertility in men or lower fertility in women, and randomization of the body plan symmetry in 50% of PCD patients) reflect the role of motile cilia in human body ^2,3^. The most cited population frequency of PCD is 1:10,000 to 1:20,000 [e.g. ^4^], but due to the heterogeneity of clinical symptoms its actual prevalence may be higher; in addition, it differs considerably depending on the population ^5,6^. PCD is mainly inherited in an autosomal recessive manner, and is characterized by a high genetic and allelic heterogeneity; until now, more than 50 genes have been identified to underlie the molecular basis of PCD ^2,7^.

*SPAG1* (sperm-associated antigen 1) is one of the genes whose recessively inherited pathogenic variants cause PCD, through impairing assembly of dynein arms, essential elements of the ciliary structure and function ^8^. Two most frequent pathogenic variants in *SPAG1*, originally reported in eight of ten ^8^ and later in eight more ^9^ unrelated Caucasian PCD patients, are: nonsense mutation in exon 16 (NM_003114.5:c.2014C>T; NP_003105.2:p.Gln672*), and a large 11,973 bp deletion, [NM_005034.4:c.61+201__NM_003114.5:c.140+1169]del, encompassing exons 1 and 2 of *SPAG1* and exons 3 and 4 of another gene, *POLR2K* (RNA Polymerase II, I and III Subunit K) located upstream of *SPAG1* in chromosome 8q22.2. Of these two variants, c.2014C>T has been described in both homozygotes and in compound heterozygotes, most frequently with the large 11,973 bp deletion as the second allele. However, no homozygotes of the large deletion have been reported. The same was observed in a much larger group of 60 *SPAG1-*PCD patients reported in our study, and in the German group of 25 *SPAG1-*PCD individuals (Heymut Omran, personal communication), prompting us to examine the basis of this unbalanced distribution of the genotypes. We suspected that the lack of homozygous patients with the large deletion reflected lethal effect of the loss of a functional POLR2K rather than of SPAG1 protein. To confirm this hypothesis we examined the effect of RNA interference (RNAi)-mediated knockdown of *POLR2K* at two levels: the whole model organism (ciliated flatworm, *Schmidtea mediterranea),* and the human cell line. *S. mediterranea* is a free-living invertebrate from phylum *Platyhelminthes* (flatworms) and it is well known for its remarkable ability to regenerate after injury or amputation thanks to the presence of numerous adult stem cells (neoblast) ^10^. Planarians are used as an alternative model system for studying the functionality of embryonic lethal genes in the adult body ^11^. They also provide an attractive model to study cilia-related genes, including PCD genes, because their locomotion (so-called gliding movement) depends on motile cilia covering the ventral site of their body. Knockdown of genes encoding ciliary proteins results in changes in the speed and locomotion pattern of worms (the so-called inchworming movement) ^12^.

1. **Materials and methods**

Patient samples collection and genetic screening

Patients were classified as PCD in concordance with European guidelines in PCD. The general clinical evaluation criteria followed the European Respiratory Society recommendations for PCD diagnosis ^13^. The majority of the patients had *situs inversus* in addition to pulmonary symptoms [see Supplementary Table 3]. Genetic data referred to in the manuscript were collected over many years as a component of routine hospital diagnostic procedures. DNA isolated form peripheral blood samples collected from affected individuals was subjected to genetic testing as earlier described ^14^. Genetic testing was a combination of single-strand conformation polymorphism (SSCP) screening, multiplex ligation-dependent probe amplification (MLPA) analysis and/or whole exome sequencing (WES); detected variants were confirmed by direct dideoxy sequencing [see Supplementary Table 4 and Sequence analysis of the pathogenic variants]. All identified pathogenic variants were examined using a variant validation tool, VariantValidator ^15^, and were classified according to the recommendation of the American College of Medical Genetics and Genomics (ACMG) and the Association for Molecular Pathology (AMP) ^16^.

*Schmidtea mediterranea*

An asexual strain of *S. mediterranea* planarian*,* originally obtained from Kerstin Bartscherer (Osnabrück University, Germany), was kindly provided by Patrick Perrigue (NanoBioMedical Centre, Adam Mickiewicz University, Poland). Planarians were maintained at 20°C (Binder cooling incubator) in water culture (1 x Montjuïch salts ^17^). Before experiments, planarians 3-5 mm in length were starved for at least 7 days.

Human cell line

The HEK293T cell line, a kind gift from Maciej Kurpisz (Institute of Human Genetics, Polish Academy of Sciences, Poland), was used. Cells were maintained in standard conditions in Dulbecco’s modified Eagle’s medium (Gibco, Thermo Fisher Scientific, Waltham, MA, USA) with 10% fetal bovine serum (Gibco, Thermo Fisher Scientific) and 1% penicillin/streptomycin solution (Sigma Aldrich, St. Louis, MO, USA), and incubated at 37°C and 5% CO_2_ in a humidified atmosphere (Binder CO_2_ incubator).

RNA interference (RNAi)-mediated knockdown in planarians

Gene knockdown was performed using double-stranded RNA (dsRNA)-feeding method, as described earlier ^18^. DsRNAs were designed based on the available transcriptomic and genomic data deposited in Planmine database ^19^, using the E-RNAi webservice ^20^ which provides information on the possible off-target effect at the dsRNA design stage. DsRNAs of *Smed-polr2k* (dd_Smed_v6_2720_0_1) and *Smed-spag1* (dd_Smed_v6_2532_0_1), hereafter referred to as *polr2k* and *spag1*, respectively), were synthetized using MEGAscript™ T7 Transcription Kit (Invitrogen). Of note, dd_Smed_v6_2532_0_1 (3145 nt) is annotated as a putative chimeric transcript in Planmine database (accessed on 06 February 2024). To prevent designing dsRNA targeting chimera, homologous transcripts of dd_Smed_v6_2532_0_1 in other *S. mediterranea* transcriptomes deposited in Planmine database were analyzed; following comparison of the homologous transcripts and predicted gene sequences, ka_Smed_v1_GCZZ01056363.1 (2315 nt) was used as a template to design and synthesize dsRNA targeting *spag1.* Transcripts to be used as a template for dsRNAs synthesis were amplified from asexual *S. mediterranea* cDNA using primers tagged with T7 RNA polymerase promotor [see Supplementary Table 5].

DsRNAs targeting *polr2k* or *spag1* were mixed with a homogenized chicken liver and administered to the worms by repetitive feeding (two times per week for 2 weeks); 10 μg dsRNA mixed with 50 μL of liver paste per 10 worms were used. As a control, worms were fed with dsRNA targeting eGFP (enhanced green fluorescence protein) prepared from the pEGFP-C1 plasmid containing eGFP insert. The plasmid was kindly provided by Maciej Śmiałek (Institute of Human Genetics, Polish Academy of Sciences, Poland). One day after the last feeding, a subgroup of worms treated with *polr2k* or *eGFP* dsRNA were cut to stimulate regeneration process.

Effects of *spag1* or *polr2k* genes knockdown on the worms phenotype were analyzed and recorded using a stereoscope (Opta-Tech, SK series) with camera. The locomotion speed of worms after *spag1* knockdown and control worms (fed with eGFP dsRNA) was measured using ImageJ ^21^ (ten worms were analyzed for each condition). Measurement of the speed of planarian’ locomotion was calculated by dividing the actual distance traveled by a worm (distance traveled minus the length of a worm) by the duration of the movie.

Transfection of HEK293T cell line

The HEK293T cells at ~ 50% confluence were separately transfected with three siRNAs (Invitrogen, Carlsbad, CA, USA) at the final concentration of 5 nM; two siRNAs targeting different regions of *POLR2K* exon 3 (Silencer™ Select siRNA, ID 10822 and 10824), and a non-targeting control (Silencer™ Select Negative Control No. 1 siRNA). Cells were transfected using jetPRIME® transfection reagent (Polyplus-transfection SA, Illkirch-Graffenstaden, France) according to the manufacturer’s protocol. Transfected cells were used for RT-qPCR, Western blot and cellular assays (cell proliferation, apoptosis).

RNA extraction and qRT-PCR in HEK293T cell line and *S. mediterranea*

Quantitative real-time reverse-transcription PCR (qRT-PCR) was carried out to confirm the effectiveness of *polr2k* and *spag1* knockdown in *S. mediterranea* or *POLR2K* knockdown in the HEK293T cell line. RNA was collected one day after the last feeding of worms or 48h after HEK293T cells transfection. Total RNA was isolated using TRI Reagent (Invitrogen) and purified from DNA contaminants using TURBO DNA-free Kit (Ambion) according to the manufacturer’s protocols. 500 ng of RNA was reverse transcribed with RevertAid H Minus First Strand cDNA Synthesis Kit (ThermoScientific), using oligo(dT) primer to process messenger RNA only. The resulting cDNA was used as a template in qRT-PCR performed using 7900HT Fast Real-Time PCR System with 96-well block module (Applied Biosystems), with HOT FIREPol® EvaGreen® qPCR Mix Plus (Solis BioDyne) and primers designed to target specific mRNAs [see Supplementary Table 5]. The level of house-keeping transcripts, Smed-*gapdh* (for worms) or ACTB (for HEK293T), was used as an endogenous control. The relative quantification of the studied genes‘ expression level was performed using a comparative CT (∆∆ CT) method. All qRT-PCR reactions were performed in three technical replicates for each biological replicate (four replicates for worms and five replicates for the cell line).

Protein analysis

48 h after transfection, cells were collected and lysed using RIPA buffer with protease inhibitor cocktail and EDTA (ThermoScientific). Protein concentration was determined using Pierce BCA Protein Assay Kit (ThermoScientific). Proteins were denatured and separated on 4-20% Mini-PROTEAN TGX Stain-free Gel (Bio Rad). After electrophoresis, proteins were transferred onto 0.45 µm PVDF Low Fluorescence membrane (Bio Rad). Membranes were blocked using 5% non-fat milk and incubated with 1:1500 anti-POLR2K (Gene-Tex, GTX132871) or 1:20000 anti-GAPDH (Abcam, ab9485). After washing, membranes were incubated with 1:40000 anti-Rabbit (Abcam, ab97051) secondary antibody. Immunoreactive protein bands were detected with Clarity Western ECL Substrate (Bio-Rad) on Chemidoc Imaging System (Bio Rad). The abundance of POLR2K was assessed in reference to GAPDH using Image Lab 6.0.1 software (Bio Rad). Each experiment was conducted in three biological replicates. Whole uncropped membrane is shown in Supplementary Figure 2.

Proliferation assay

Cells were seeded on 96-well plate (40,000 cells per well) in culture medium and were transfected 24h after seeding. The CCK8 test (Cell Counting Kit 8, Sigma Aldrich) was conducted in a time-dependent manner from 0 to 60 h after transfection. Cells were incubated with 10 μL of CCK8 reagent for 4 h at 37◦C. Absorbance was measured using GloMax-Multi+ Detection System (Promega). Each experiment was conducted in four technical and three biological replicates.

Apoptosis assay

Cells were seeded on 12-well plate (200,000 cells per well) in culture medium and were transfected 24h after seeding. 48 h after transfection, cells were collected and stained with Annexin V-FITC (BD Biosciences, San Jose, CA, USA). Populations of apoptotic and non-apoptotic cells were detected with a CytoFlex S cytometer (Beckman Coulter, Brea, CA, USA). Results were analyzed using CytExpert Software (Beckman Coulter). Each experiment was conducted in three biological replicates. Representative plots for apoptosis assay are shown in Supplementary Figure 3.

Statistical analysis

The statistical significance of differences between the proportion of genotypes observed and expected under Hardy-Weinberg equilibrium was calculated using chi-square test.

In the analysis of the relative gene expression level, apoptosis or planarians’ locomotion speed, where two independent means were compared, data were first analyzed for the normality with Shapiro–Wilk test, and statistical significance of the difference was calculated using unpaired two-tailed t-test.

In proliferation assays, the statistical significance of the differences between control and *POLR2K* siRNAs-treated cells was calculated with a two-way ANOVA using the Tukey post-hoc test. The statistical significance of the results was evaluated and visualized using the GraphPad Prism version 9 (GraphPad Software).

1. **Additional Discussion**

The lack of homozygotes of the large deletion encompassing parts of *SPAG1* and the upstream *POLR2K* has been observed in the earlier studies ^8,9^, but was not as obvious due to the smaller number of reported unrelated Caucasian PCD patients with pathogenic *SPAG1* variants (n=18). In 60 unrelated Polish-Slovakian patients with two *SPAG1* mutations, as well as in the German group of 25 patients, the lack of homozygotes with the large deletion was striking. The expected number of homozygotes under Hardy-Weinberg equilibrium in the combined group of 103 Caucasian SPAG1-PCD patients was eight, while none was observed; this discrepancy between the observed and expected distribution of haplotypes was highly significant (p=0.0066). A similar distribution of genotypes, with the significant lack of homozygotes of one of the alleles, has been described in *PMM2* gene. In the cohort of 54 Caucasian patients suffering with carbohydrate-deficient–glycoprotein syndrome type 1 with a PMM deficiency, 43 were heterozygous for R141H variant, but no homozygotes were observed in contrast to 8 expected under Hardy-Weinberg equilibrium; the authors concluded that this distribution was probably caused by selection against the homozygous R141H ^22^ .

It should be emphasized that the frequency of homozygous mutations introducing premature STOP codon located in *SPAG1* exon 16 (c.2014C>T) was even higher than expected under equilibrium, indicating the lack of selection against a homozygous deficiency of the wild-type *SPAG1*. The possibility that homozygous truncation at exon 16 was less deleterious than the lack of the full-length protein can be rejected based on the observation that the compound heterozygote, in whom the large deletion was combined with the pathogenic variant in exon 2 (c.1A>G, expected to abrogate the start codon and thus to prevent translation of the whole protein), had a typical PCD phenotype. We hypothesized that the lack of homozygotes with the large deletion, encompassing exons 1-2 of *SPAG1* and exons 3-4 of *POLR2K*, reflected a highly deleterious, possibly lethal effect of the loss of a functional POLR2K rather than of SPAG1 protein.

To provide a molecular context to this observation, we analyzed the impact of *POLR2K* silencing on phenotypes at the whole organism (planarians) and cellular (human cell line) levels. The dsRNA-mediated knockdown of *spag1* in the *S. mediterranea* model did not affect viability of the organisms, but – as expected – impaired worms movement, confirming the evolutionary conserved role of *SPAG1* in the motile cilia function, which was also previously demonstrated using a vertebrate model, zebrafish ^8^. In contrast, silencing of *polr2k* in the same model resulted in a much more severe phenotype, and suggested that *polr2k* plays an essential role in maintaining body homeostasis by regulating cell turnover, probably through the impact on planarian stem cells proliferation. Our observations in the planarian model are in line with those in other studies where the deleterious/lethal effects of the knockdown of other essential genes on *S. mediterranea* phenotype have been reported [e.g. ^11,23–27^]. For example, RNAi-mediated knockdown of the essential gene, *Argonaute RISC Catalytic Component 2* (*Smed-ago2*), led to regeneration defects or tissue regression in worms, ultimately causing their death, demonstrating that *Smed-ago2* is crucial for tissue homeostasis and regeneration and acts through a regulation of adult stem cells proliferation ^24^.

Our results obtained in the human cell culture with siRNA-mediated knockdown of *POLR2K* shed light on the mechanisms of defects observed in the planarian model, confirming essential role of *POLR2K* in the regulation of vital cell functions, manifested by decreased cell proliferation and increased apoptosis upon its knockdown.

The essential role of *POLR2K* is supported by many studies. *POLR2K* represents one of the smallest subunits of RNA polymerase II, shared with two other RNA polymerases; in consequence, *POLR2K* is involved in the RNA processing machinery and improves RNA polymerase III pre-initiation complex assembly ^28^. There are reports about the association of this gene with cancer development ^29,30^, lethal prostate cancer ^31^, and predicted breast cancer immunotherapy ^32^. Other studies indicate protective role of *POLR2K* against liver injury ^33^ and its participation in lymphocyte innateness ^34^. In a few reports *POLR2K* has been mentioned among hub genes responsible for diverse gene interaction networks: in chronic kidney disease ^35^, polycystic ovary syndrome ^36^, cancer ^37^, high-altitude hypoxia ^28^. In the study, which demonstrated that the high mortality of bovine embryos derived from somatic cell nuclear transfer is related to the absence of three regulatory networks in the early embryonic stage, *POLR2K* has been mentioned in the core of one of these networks ^38^.

In spite of the repetitive reports suggesting the importance of *POLR2K* in many cellular processes, which is expected given the role of POLR2K in RNA processing machinery, the direct observation of phenotype changes after RNAi-mediated *POLR2K* knockdown in a model organism or a human cell line has not been published so far. Our findings confirm that *POLR2K* is necessary for functioning of living organisms and suggest that its influence on cell turnover is exerted through a regulation of cell proliferation and protection from cell apoptosis. Moreover, our study provides evidence that the essential role of *POLR2K* in vital cell functions is evolutionary conserved. Finally, these results, together with the aforementioned studies regarding *POLR2K* as a hub gene involved in many essential interactions, support our starting hypothesis that the lack of homozygotes of the large deletion involving *SPAG1* and *POLR2K* genes is caused by the lethal impact of the POLR2K deficiency.

In view of the fundamental role of *POLR2K* in the normal functioning of the cell, it is tempting to hypothesize that its absence is lethal. Similar to earlier suggestions concerning the missing variant in the *PMM2* gene^22^, it would be interesting to look for the association of the homozygous large deletion variant encompassing *POLR2K* and *SPAG1* with idiopathic miscarriages; however, the expected frequency of families, in which both parents are carriers of the large deletion, is very low. Alternatively, analyzing progeny of a transgenic animal model (e.g. mouse or fish) carrying the heterogenic large deletion may be the way to confirm a possible lethality of the homozygous deficiency of *POLR2K*.

1. **Supplementary Tables**

**Supplementary Table 1.** **Description of pathogenic variants.**

| **Affected *SPAG1* exons** | **Pathogenic variants** (genome build: GRCh38) | **Evidence of pathogenicity** | **ACMG/AMP**  **category** |
| --- | --- | --- | --- |
| 1_2 | large (11,973bp) deletion, encompassing:  chr8(GRCh38):g.100151617_100163589del ^nsv3873246^,  corresponding to  [NM_005034.4:c.61+201 (*POLR2K* intron 2)_ NM_003114.5:c.140+1169 (*SPAG1* intron 2)]del | very strong | PVS1 null variant - multiexonic deletion |
| 2 | NM_003114.5:c.1A>G;  NP_003105.2:p.Met1 loss of start or splice region variant ^novel^ | very strong | PVS1 null variant - initiation codon |
| 7 | NM_003114.5:c.600_603del;  NP_003105.2:p.T201Rfs*28 ^novel; rs1258068273^ | very strong | PVS1 null variant - frameshift |
| 8 | NM_003114.5:c.762_763insAAAA  NP_003105.2:p.L255Kfs*7 ^novel^ | very strong | PVS1 null variant - frameshift |
| 11 | NM_003114.5:c.1395del;  p.G466Afs*21  ^novel^ | very strong | PVS1 null variant - frameshift |
| 12 | NM_003114.5:c.1436G>A;  NP_003105.2:p.G479E; acceptor splice site region ^novel; rs1817848640^ | strong | PS3 acceptor splice site region – experiment confirmed damaging effect on the gene product^1^ |
| 16 | NM_003114.5:c.2014C>T;  NP_003105.2:p.Q672* ^rs201740530^ | very strong | PVS1 null variant - nonsense |

Available rs numbers are indicated in superscript; “novel” superscript indicates pathogenic *SPAG1* variants not reported before in the context of PCD; ^1^the disruption of acceptor splice site was confirmed at the mRNA level [Supplementary Figure 4].

**Supplementary Table 2. Distribution of patients’ genotypes in the analyzed group of 60 *SPAG1*-PCD patients**.

| **Observed genotypes (affected *SPAG1* exons)** | **Number of unrelated individuals with a particular genotype** |
| --- | --- |
| 1_2/16 | 28 (including 1 Slovak) |
| 16/16 | 27 (including 6 Slovaks) |
| 16/8 | 1 |
| 16/11 | 1 |
| 16/12 | 1 |
| 1_2/7 | 1 |
| 1_2/2 | 1 |

**Supplementary Table 3. Number of patients with and without *situs inversus.***

| **Observed genotypes (affected *SPAG1* exons)** | **Number of patients with the presence/absence of situs inversus (SI)** |
| --- | --- |
| 1_2/16 | 20 with SI; 5 without SI; 3 N/A |
| 16/16 | 16 with SI; 10 without SI; 1 N/A |
| 16/8 | 1 without SI |
| 16/11 | 1 without SI |
| 16/12 | 1 without SI |
| 1_2/7 | 1 with SI |
| 1_2/2 | 1 without SI |

N/A- not applicable; no information about SI in medical information.

**Supplementary Table 4. List of primers used for patients samples analysis.**

| **Primers name** | **Primer sequence** | **Expected product size [bp]** |
| --- | --- | --- |
| POLR2K_i2_F | tagacctgggttcgtccttg | in sample with deletion:  311 |
| SPAG1_i2_R | tttttatttatccgtccttgacc |  |
| SPAG1_2_F | tggttaccactatccaaattgag | 321 |
| SPAG1_2_R | catgcatgggcaaaaaca |  |
| SPAG1_7_F | cctaagcttggggaacctttt | 281 |
| SPAG1_7_R | aaaccccaacaaacatctgc |  |
| SPAG1_8_F | accttaggcttgcttgttgc | 229 |
| SPAG1_8_R | tggtctttattaatggatcctgc |  |
| SPAG1_11B_F2 | ctgaagagccagggcaac | 201 |
| SPAG1_11B_R2 | gcgcctgtcattcagga |  |
| SPAG1_12_F | ggccgtcttgtaattctggt | 247 |
| SPAG1_12_R | ggatcttggatattgcattcac |  |
| SPAG1_16_F | acagatagccaatctttacctttca | 277 |
| SPAG1_16_R | cctcaatcccatcccaagat |  |
| *Primers for cDNA analysis* | | |
| SPAG1_e11_F | CAAGTACTCGGCGGCAAT | 180 |
| SPAG1_e13_R | CGCCTCAGAAGAGGTTTCAT |  |
| GAPDH_e4_F | ACGGGAAGCTTGTCATCAAT | 382 |
| GAPDH_e8_R | GGGCCATCCACAGTCTTCT |  |

Lower case – primers in introns; upper case – primers in exons

**Supplementary Table 5. List of primers used for dsRNA synthesis and in RT-qPCR**

| **Primers name** | **Primer sequence** |
| --- | --- |
| *Primers for dsRNA synthesis* |  |
| dsRNA_Smed_POLR2K_R | TAATACGACTCACTATAGGGcaacacttcaacgaccttcaaa |
| dsRNA_Smed_POLR2K_F | TAATACGACTCACTATAGGGttcgtctgaagcaaaacaagt |
| dsRNA_Smed_SPAG1_R | TAATACGACTCACTATAGGGttctttttctaacacggcagc |
| dsRNA_Smed_SPAG1_F | TAATACGACTCACTATAGGGaattgccgacgaacttgaga |
| dsRNA_eGFP_R | TAATACGACTCACTATAGGGagttcaccttgatgccgttc |
| dsRNA_eGFP_F | TAATACGACTCACTATAGGGcacatgaagcagcacgactt |
| *Primers for RT-qPCR of worms (Smed)  and human (Hs) samples* |  |
| RTPCR_Smed_POLR2K_R | ataatgtctttaggcttcatttcg |
| RTPCR_Smed_POLR2K _F | tgaataattcgtctgaagcaaaac |
| RTPCR_Smed_SPAG1_R | tccccatttcccgatagcat |
| RTPCR_Smed_SPAG1_F | cgattgcaaaagttgtcgcc |
| RTPCR_Smed_GAPDH_R | cagatcgattacacggcaac |
| RTPCR_Smed_GAPDH_F | atcaaggccgctattaagca |
| RTPCR_Hs_POLR2K_R | cccagcattcatcgagcatc |
| RTPCR_Hs_POLR2K_F | cctccaaagcagcaaccaat |
| RTPCR_Hs_ACTB_R | atcttgatcttcattgtgctg |
| RTPCR_Hs_ACTB_F | cttcctgggcatggagtcc |

The uppercase letters describe the sequence recognized by the T7 RNA polymerase.

1. **Supplementary Figures**


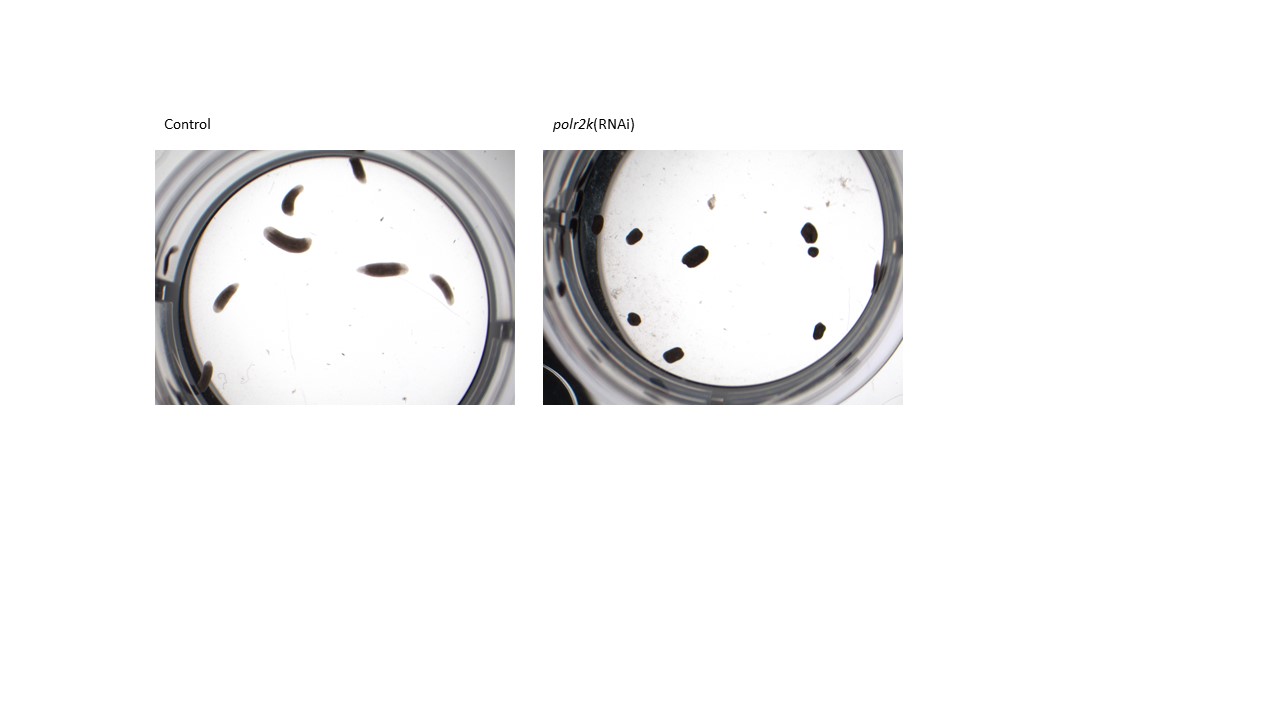


**Supplementary Figure 1.** Analysis of the effect of dsRNA-mediated *polr2k* knockdown on the regeneration capacity of *S. mediterranea;* an example of *polr2k*(RNAi) worms regenerated from the middle part of worm in comparison to a control group at the same time point (7 days post amputation, dpa); the presented defects of the regeneration process ultimately lead to the death of *polr2k*-silenced worms.


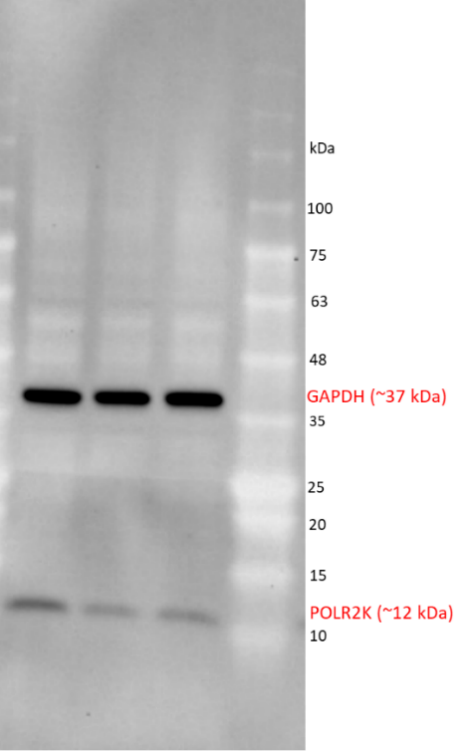


**Supplementary Figure 2.** Chemiluminescent blot for Western Blot of POLR2K and GAPDH proteins in HEK293T cell line after *POLR2K* gene silencing.


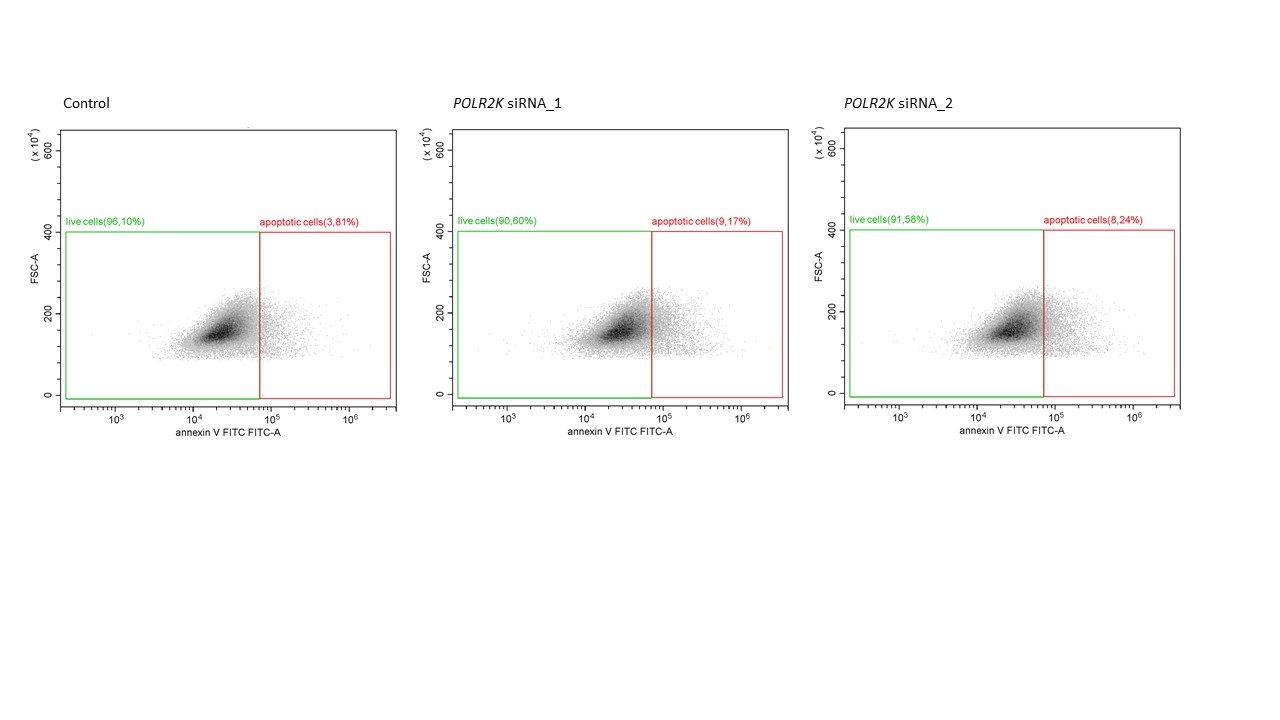


**Supplementary Figure 3** Representative plots for apoptosis assay of HEK293T cells after *POLR2K* siRNA-mediated knockdown.

**
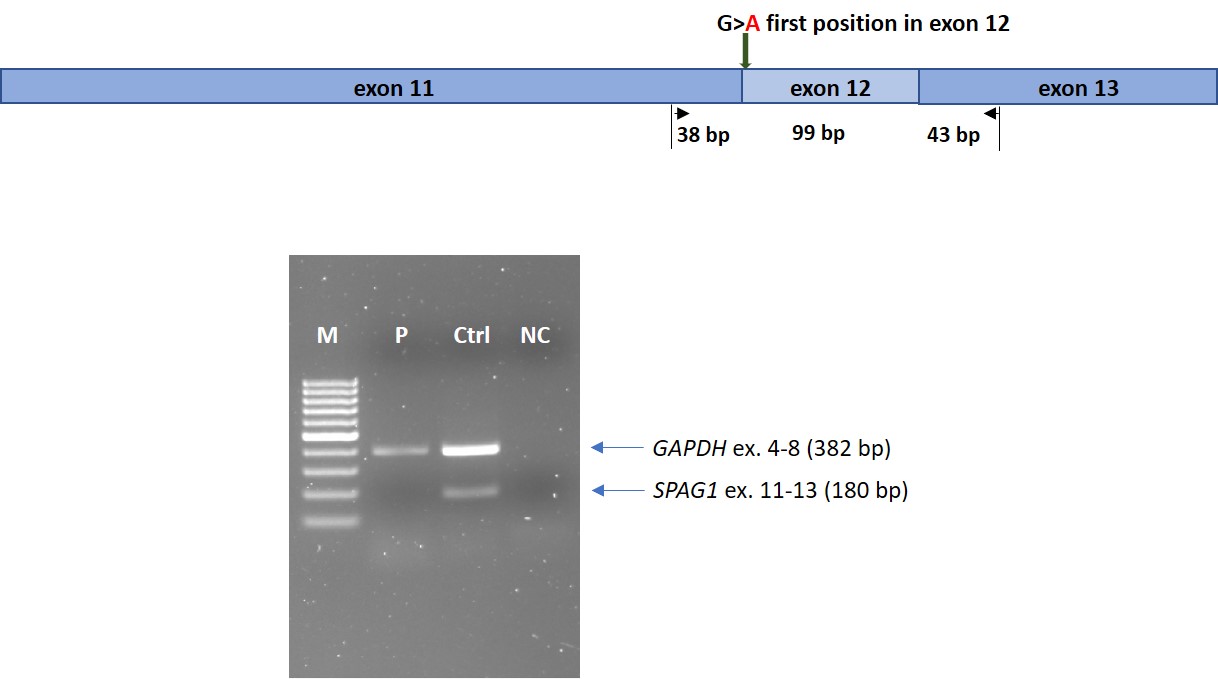
**

**Supplementary Figure 4.** Analysis of the effect of a substitution at the first position of exon 12 of *SPAG1.* The G>A substitution at the first position of exon 12 (theoretically causing a missense mutation, p.G479E) from patient’s cDNA disrupted acceptor splicing site, resulting in the lack of a product amplified with primers located in exons 11 and 13. Legend: M- marker DNA Molecular Weight Marker, 100bp Ladder (Thermofisher), P- patient, Ctrl- healthy control (non-PCD individual), NC- negative control (water).

1. **Sequence analysis of the pathogenic variants**

***POLR2K* exons 3_4 - *SPAG1* exons 1_2**

Only sequence of the allele with the large deletion was analyzable using Sanger sequencing

chr8(GRCh38):g.100151617_100163589del; breakpoint: in *POLR2K* intron 2 (c.61+201) and *SPAG1* intron 2 (c.140+1169);

**large deletion**


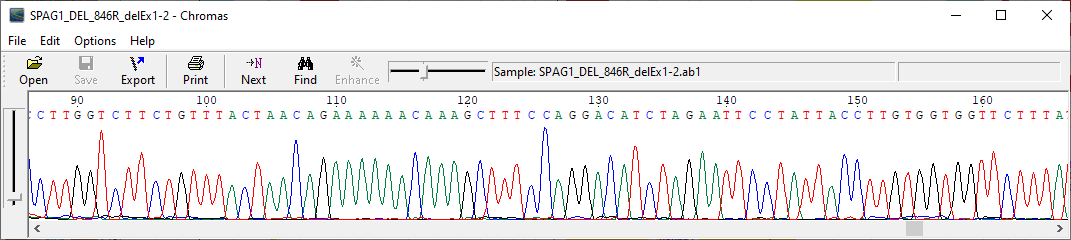


*POLR2K* intron 2..*.SPAG1* intron2

**AACAGAAAAAAACAAAGCTcgttgagatttcattctctctcctgga…cacttgtggagcatgaactgctttgctTTCCAGGACATCTAGAATT**

Uppercase: the sequence in the individual with the large deletion.

***SPAG1* exon 2**

**wt**


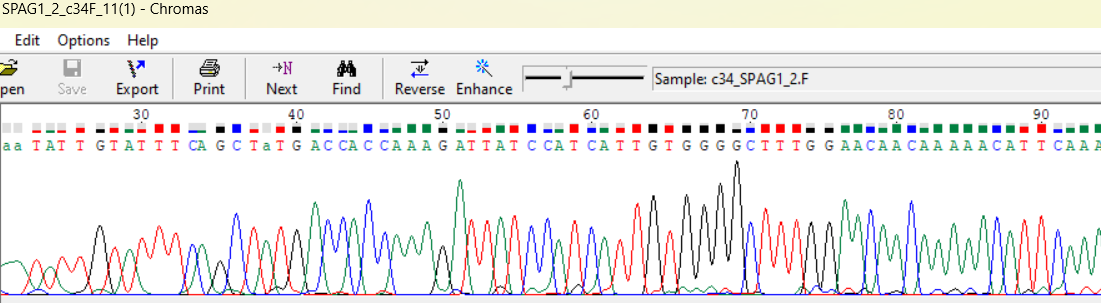


**NM_003114.5:c.1A>G**


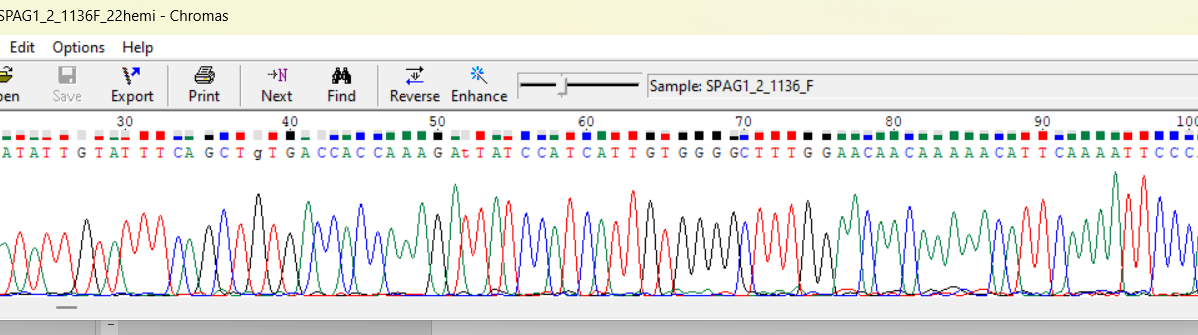


**hemizygote**

***SPAG1* exon 7**

**wt**


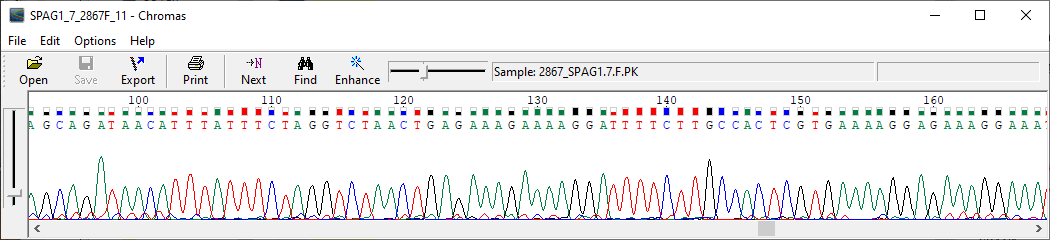


**NM_003114.5:c.600_603delCTAA** **heterozygote**


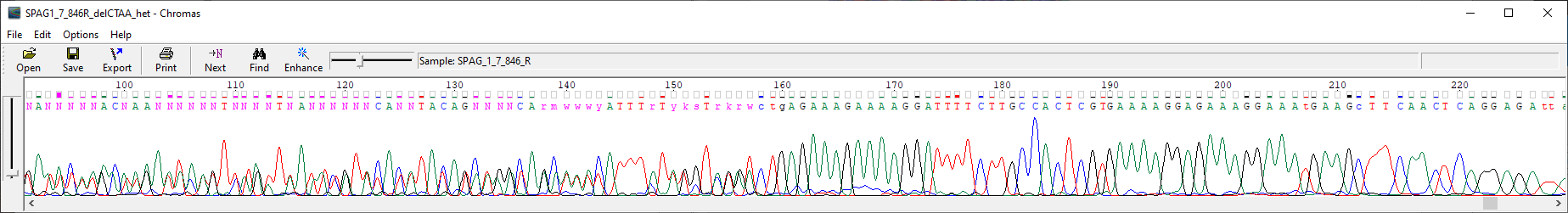


***SPAG1* exon 8**

**wt**


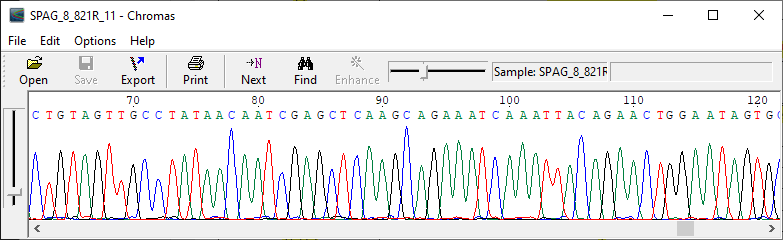


**NM_003114.5:c.762_763insAAAA** **heterozygote**


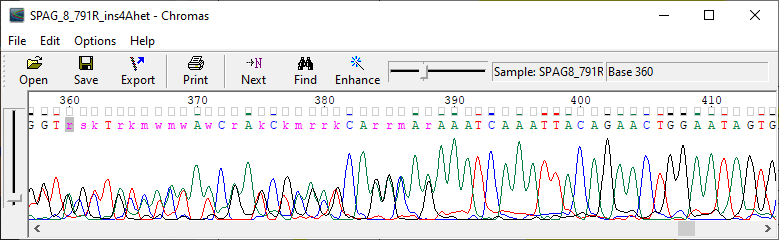


***SPAG1* exon 11**

**wt**


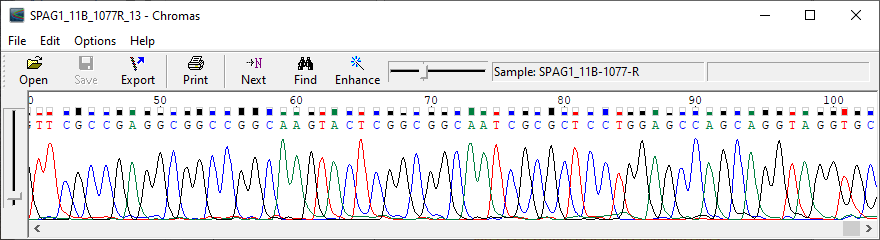


**NM_003114.5:c.1395delC**


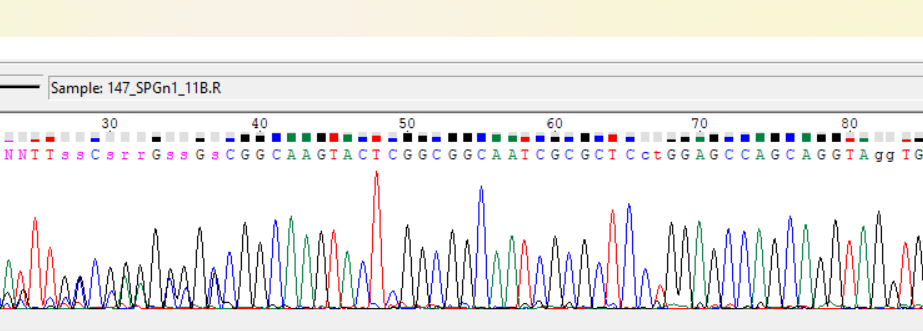


**heterozygote**

***SPAG1* exon 12**

**wt**


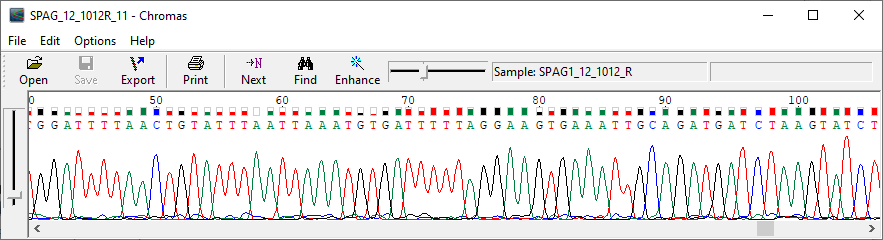


**NM_003114.5:c.1436G>A**


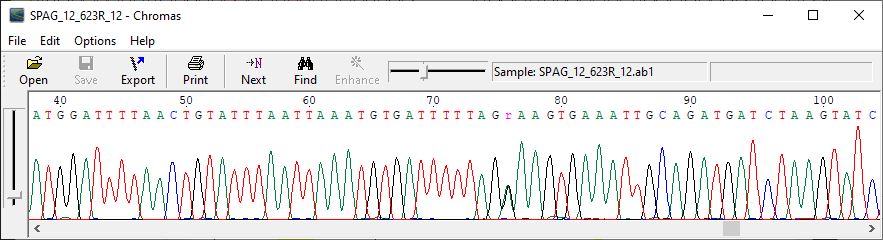


**heterozygote**

***SPAG1* exon 16**

**wt**


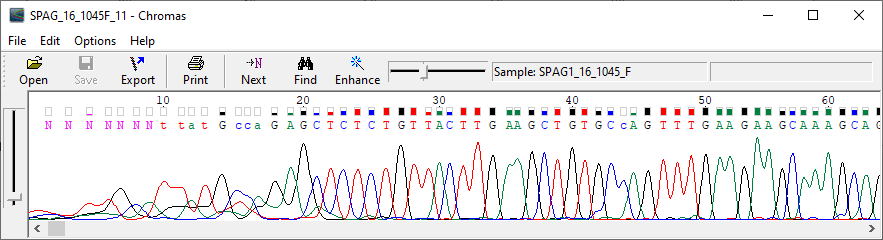


**NM_003114.5:c.2014C>T**


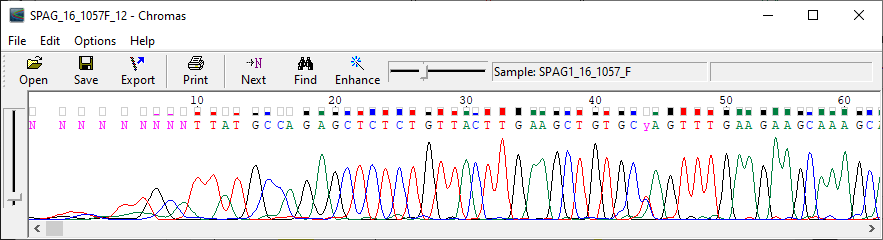


**heterozygote**

**NM_003114.5:c.2014C>T**


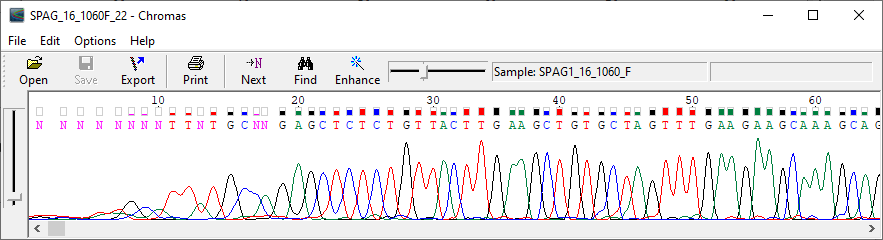


**homozygote**

**Supplementary References**

1. Wirschell M, Yamamoto R, Alford L, Gokhale A, Gaillard A, Sale WS. Regulation of ciliary motility: conserved protein kinases and phosphatases are targeted and anchored in the ciliary axoneme. *Arch Biochem Biophys*. 2011;510(2):93-100. doi:10.1016/j.abb.2011.04.003

2. Goutaki M, Shoemark A. Diagnosis of Primary Ciliary Dyskinesia. *Clin Chest Med*. 2022;43(1):127-140. doi:10.1016/j.ccm.2021.11.008

3. Wallmeier J, Nielsen KG, Kuehni CE, et al. Motile ciliopathies. *Nat Rev Dis Primers*. 2020;6(1):77. doi:10.1038/s41572-020-0209-6

4. Wee WB, Gatt D, Seidl E, Santyr G, To T, Dell SD. Estimates of primary ciliary dyskinesia prevalence: a scoping review. *ERJ Open Res*. 2024;10(4):00989-02023. doi:10.1183/23120541.00989-2023

5. Ardura-Garcia C, Goutaki M, Carr SB, et al. Registries and collaborative studies for primary ciliary dyskinesia in Europe. *ERJ Open Res*. 2020;6(2):00005-02020. doi:10.1183/23120541.00005-2020

6. Hannah WB, Seifert BA, Truty R, et al. The global prevalence and ethnic heterogeneity of primary ciliary dyskinesia gene variants: a genetic database analysis. *Lancet Respir Med*. 2022;10(5):459-468. doi:10.1016/S2213-2600(21)00453-7

7. Legendre M, Zaragosi LE, Mitchison HM. Motile cilia and airway disease. *Semin Cell Dev Biol*. 2021;110:19-33. doi:10.1016/j.semcdb.2020.11.007

8. Knowles MR, Ostrowski LE, Loges NT, et al. Mutations in SPAG1 cause primary ciliary dyskinesia associated with defective outer and inner dynein arms. *Am J Hum Genet*. 2013;93(4):711-720. doi:10.1016/j.ajhg.2013.07.025

9. Djakow J, Kramná L, Dušátková L, et al. An effective combination of sanger and next generation sequencing in diagnostics of primary ciliary dyskinesia. *Pediatr Pulmonol*. 2016;51(5):498-509. doi:10.1002/ppul.23261

10. Reddien PW. The Cellular and Molecular Basis for Planarian Regeneration. *Cell*. 2018;175(2):327-345. doi:10.1016/j.cell.2018.09.021

11. Ziman B, Barghouth PG, Maciel EI, Oviedo NJ. TRAF-like Proteins Regulate Cellular Survival in the Planarian Schmidtea mediterranea. *iScience*. 2020;23(11):101665. doi:10.1016/j.isci.2020.101665

12. Rabiasz A, Ziętkiewicz E. Schmidtea mediterranea as a Model Organism to Study the Molecular Background of Human Motile Ciliopathies. *Int J Mol Sci*. 2023;24(5):4472. doi:10.3390/ijms24054472

13. Lucas JS, Barbato A, Collins SA, et al. European Respiratory Society guidelines for the diagnosis of primary ciliary dyskinesia. *Eur Respir J*. 2017;49(1):1601090. doi:10.1183/13993003.01090-2016

14. Zietkiewicz E, Bukowy-Bieryllo Z, Rabiasz A, et al. CFAP300: Mutations in Slavic Patients with Primary Ciliary Dyskinesia and a Role in Ciliary Dynein Arms Trafficking. *Am J Respir Cell Mol Biol*. 2019;61(4):440-449. doi:10.1165/rcmb.2018-0260OC

15. Freeman PJ, Hart RK, Gretton LJ, Brookes AJ, Dalgleish R. VariantValidator: Accurate validation, mapping, and formatting of sequence variation descriptions. *Hum Mutat*. 2018;39(1):61-68. doi:10.1002/humu.23348

16. Richards S, Aziz N, Bale S, et al. Standards and guidelines for the interpretation of sequence variants: a joint consensus recommendation of the American College of Medical Genetics and Genomics and the Association for Molecular Pathology. *Genet Med*. 2015;17(5):405-424. doi:10.1038/gim.2015.30

17. Cebrià F, Newmark PA. Planarian homologs of netrin and netrin receptor are required for proper regeneration of the central nervous system and the maintenance of nervous system architecture. *Development*. 2005;132(16):3691-3703. doi:10.1242/dev.01941

18. Rouhana L, Weiss JA, Forsthoefel DJ, et al. RNA interference by feeding in vitro-synthesized double-stranded RNA to planarians: methodology and dynamics. *Dev Dyn*. 2013;242(6):718-730. doi:10.1002/dvdy.23950

19. Rozanski A, Moon H, Brandl H, et al. PlanMine 3.0-improvements to a mineable resource of flatworm biology and biodiversity. *Nucleic Acids Res*. 2019;47(D1):D812-D820. doi:10.1093/nar/gky1070

20. Horn T, Boutros M. E-RNAi: a web application for the multi-species design of RNAi reagents—2010 update. *Nucleic Acids Res*. 2010;38(Web Server issue):W332-W339. doi:10.1093/nar/gkq317

21. Rompolas P, Azimzadeh J, Marshall WF, King SM. Analysis of ciliary assembly and function in planaria. *Methods Enzymol*. 2013;525:245-264. doi:10.1016/B978-0-12-397944-5.00012-2

22. Matthijs G, Schollen E, Van Schaftingen E, Cassiman JJ, Jaeken J. Lack of homozygotes for the most frequent disease allele in carbohydrate-deficient glycoprotein syndrome type 1A. *Am J Hum Genet*. 1998;62(3):542-550.

23. Palakodeti D, Smielewska M, Lu YC, Yeo GW, Graveley BR. The PIWI proteins SMEDWI-2 and SMEDWI-3 are required for stem cell function and piRNA expression in planarians. *RNA*. 2008;14(6):1174-1186. doi:10.1261/rna.1085008

24. Li YQ, Zeng A, Han XS, et al. Argonaute-2 regulates the proliferation of adult stem cells in planarian. *Cell Res*. 2011;21(12):1750-1754. doi:10.1038/cr.2011.151

25. Thiruvalluvan M, Barghouth PG, Tsur A, Broday L, Oviedo NJ. SUMOylation controls stem cell proliferation and regional cell death through Hedgehog signaling in planarians. *Cell Mol Life Sci*. 2018;75(7):1285-1301. doi:10.1007/s00018-017-2697-4

26. Pryszlak M, Wiggans M, Chen X, et al. The DEAD-box helicase DDX56 is a conserved stemness regulator in normal and cancer stem cells. *Cell Rep*. 2021;34(13):108903. doi:10.1016/j.celrep.2021.108903

27. Allen JM, Balagtas M, Barajas E, et al. RNAi Screen of RING/U-Box Domain Ubiquitin Ligases Identifies Critical Regulators of Tissue Regeneration in Planarians. *Frontiers in Cell and Developmental Biology*. 2022;9. Accessed February 25, 2024. https://www.frontiersin.org/articles/10.3389/fcell.2021.803419

28. Li Q, Xu Z, Fang F, Shen Y, Lei H, Shen X. Identification of key pathways, genes and immune cell infiltration in hypoxia of high-altitude acclimatization via meta-analysis and integrated bioinformatics analysis. *Front Genet*. 2023;14:1055372. doi:10.3389/fgene.2023.1055372

29. Heidenblad M, Lindgren D, Jonson T, et al. Tiling resolution array CGH and high density expression profiling of urothelial carcinomas delineate genomic amplicons and candidate target genes specific for advanced tumors. *BMC Med Genomics*. 2008;1:3. doi:10.1186/1755-8794-1-3

30. Lin Y, Li Z, Ozsolak F, et al. An in-depth map of polyadenylation sites in cancer. *Nucleic Acids Res*. 2012;40(17):8460-8471. doi:10.1093/nar/gks637

31. Kelly RS, Sinnott JA, Rider JR, et al. The role of tumor metabolism as a driver of prostate cancer progression and lethal disease: results from a nested case-control study. *Cancer Metab*. 2016;4:22. doi:10.1186/s40170-016-0161-9

32. López-Cortés A, Cabrera-Andrade A, Vázquez-Naya JM, et al. Prediction of breast cancer proteins involved in immunotherapy, metastasis, and RNA-binding using molecular descriptors and artificial neural networks. *Sci Rep*. 2020;10(1):8515. doi:10.1038/s41598-020-65584-y

33. Pang C, Sheng Y chen, Jiang P, Wei H, Ji L li. Chlorogenic acid prevents acetaminophen-induced liver injury: the involvement of CYP450 metabolic enzymes and some antioxidant signals. *J Zhejiang Univ Sci B*. 2015;16(7):602-610. doi:10.1631/jzus.B1400346

34. Gutierrez-Arcelus M, Teslovich N, Mola AR, et al. Lymphocyte innateness defined by transcriptional states reflects a balance between proliferation and effector functions. *Nat Commun*. 2019;10(1):687. doi:10.1038/s41467-019-08604-4

35. Xia J, Hou Y, Cai A, et al. An integrated co-expression network analysis reveals novel genetic biomarkers for immune cell infiltration in chronic kidney disease. *Front Immunol*. 2023;14:1129524. doi:10.3389/fimmu.2023.1129524

36. Devarbhavi P, Telang L, Vastrad B, Tengli A, Vastrad C, Kotturshetti I. Identification of key pathways and genes in polycystic ovary syndrome via integrated bioinformatics analysis and prediction of small therapeutic molecules. *Reprod Biol Endocrinol*. 2021;19(1):31. doi:10.1186/s12958-021-00706-3

37. Zhang WJ, Yue KL, Wang JZ, Zhang Y. Association between heat shock factor protein 4 methylation and colorectal cancer risk and potential molecular mechanisms: A bioinformatics study. *World J Gastrointest Oncol*. 2023;15(12):2150-2168. doi:10.4251/wjgo.v15.i12.2150

38. Zhao L, Long C, Zhao G, et al. Reprogramming barriers in bovine cells nuclear transfer revealed by single‐cell RNA‐seq analysis. *J Cell Mol Med*. 2022;26(18):4792-4804. doi:10.1111/jcmm.17505
